# A Review of 2008 for *PLoS Computational Biology*

**DOI:** 10.1371/journal.pcbi.1000275

**Published:** 2009-01-30

**Authors:** Evie Browne, Rosemary Dickin, Philip E. Bourne

**Affiliations:** 1 San Diego Supercomputer Center, University of California San Diego, La Jolla, California, United States of America; 2 Skaggs School of Pharmacy and Pharmaceutical Sciences, University of California San Diego, La Jolla, California, United States of America

A very Happy New Year to all our authors, readers, editors, and reviewers from everyone at the Public Library of Science! 2008 was a remarkable year for *PLoS Computational Biology*; which saw 50% more submissions than in 2007 (900 full articles and 175 presubmission inquiries), more than 260 high-quality research articles published, and regular contributions of Editorials, Reviews and Perspectives, and Education and Society pages. This growth and maturity of content leaves no doubt that our Journal has become a leading reference for the field of computational biology and a trusted place to publish.

Such success has come through the hard work of our Editors, not only from our Editorial Board but also from the anonymous reviewers and Guest Editors who expend so much time and energy in the assessment of submitted manuscripts (each averaging 2.8 reviews and 1 to 2 rounds of revisions), and from the attention to detail and care taken over the content.

Peer review by external experts is essential to ensuring that the work published in *PLoS Computational Biology* is of the very highest quality, and we are grateful to all of our reviewers for their thoughtful and informed comments. Guest Editors are those who step in to edit one particular paper that describes work in an area of research that falls outside the expertise of the more than 50 volunteer Editors on our Board. The flexibility and availability of these Guest Editors is invaluable in our being able to provide a high level of review, as well as playing an important role in maintaining the broad appeal and vibrancy of the Journal. Their names can be found together in Table S1 as an acknowledgment of the good work they do and the time they donate to improve the body of scientific literature and knowledge.

In 2008, our pool of reviewers included approximately 1,300 scientists in 36 countries, including Vietnam, Mexico, Brazil, and Afghanistan, as well as in countries such as Israel, Germany, and Japan, where the Journal is better-known. This impressive geographical spread indicates that we are reaching the best of the best across the scientific world, something only a well-respected journal of quality is able to accomplish.

Organic growth requires that we constantly assess both the kinds of papers we accept and the standards of research they represent. We have revised our scope statement to reflect slight changes in our focus (see http://www.ploscompbiol.org/static/information.action), and we constantly refine our Editorial Board (http://www.ploscompbiol.org/static/edboard.action) to handle the number and types of papers we are encouraging. Experiencing solid growth can come at a price to the speed of our Editorial processes, however, and while we aim to provide a decision to our authors within 35 days, some papers defy this time limit. We are confident, however, that with your continued help and support, we will reach our targets more consistently this year. As authors, you appreciate a swift response time, and as reviewers you can help us achieve this by making a commitment in 2009 to return reviews within two weeks.

Looking ahead in 2009, you can expect to see not only more great research, but also greater connectivity between content found in different PLoS journals and among members of your community. As an example of the former, *PLoS Computational Biology* will be working with *PLoS ONE* to feature developments in software important to our discipline. For the latter, the community can read and participate in discussions that start when readers post a comment or rating on a published article.

As we have done since our launch, we welcome your feedback on how we're doing and what we should be doing going forward. This is your Journal, and our open philosophy encourages your engagement in it. By working together, we can further establish the importance of our science to our understanding of living systems and make a positive contribution to moving it forward even in these uncertain times.

Once again, many thanks to all of you for your support and commitment to making 2008 a successful year for *PLoS Computational Biology* and to ensuring that we are able to achieve even more in the upcoming year.

## Supporting Information

Table S1 Guest Editors and Reviewers for *PLoS Computational Biology* in 2008
(0.18 MB PDF)

