# Supplementary material for: A Review of 2008 for PLoS Computational Biology
Source: PLoS Comput Biol. 2009 Jan 30;5(1):e1000275. doi: 10.1371/journal.pcbi.1000275 (PMC2613517; doi:10.1371/journal.pcbi.1000275)
Supplement: Table S1 — Guest Editors and Reviewers for PLoS Computational Biology in 2008 (0.18 MB PDF) [file pcbi.1000275.s001.pdf]

PLoS Computational Biology Guest Editors 2008

Aloy, Patrick	Hartley, Stephen	Serrano, Luis
Baker, Nathan	Hensel, Reinhard	Shimizu, Tom
Banga, Julio	Hogeweg, Paulien	Shvartsman, Stanislav
Bashford, Jim	Hunt, C. Anthony	Sidow, Arend
Benham, Craig	Hurst, Laurence	Sjolander, Kimmen
Berg, Johannes	Ito, Keita	Sprang, Stephen
Boccaletti, Stefano	Jensen, Mark	Sukharev, Sergei
Bonfils, Claude	Kaibuchi, Kozo	Tang, Chao
Brent, Michael	Kannan, Natarajan	Teichmann, Sarah
Brookmeyer, Ron	Kortemme, Tanja	Tieleman, Peter
Burch, Christina	Krogan, Nevan	Vajda, Sandor
Bussemaker, Harmen	Kuhlman, Brian	Verkhivker, Gennady
Butler, Peter J.	Lauffenburger, Douglas	Vogel, Christine
Bystroff, Chris	Lin, Simon	Warshel, Arieh
Chait, Brian	Long, Glenis	Weinreich, Daniel
Chothia, Cyrus	Loomis, William	Workman, Christopher
Cohen, Mitchell	Maini, Philip	Xing, Eric
Couzin, Iain	Marti-Renom, Marc	You, Lingchong
Cowburn, David	McCulloch, Andrew	Zhang, Jianzhi
Czirók, András	Michailova, Anushka	Zhang, Zhaolei
Daniel, Tom	Michor, Franziska	Zhaoping, Li
Davenport, Miles	Morris, Jeff	Zhou, Jizhong
Deem, Michael	Mukherjee, Sayan	Zweckstetter, Markus
Dunker, Keith	Nash, Martyn	
Elber, Ron	Orengo, Christine	
Elcock, Adrian	Pettitt, B. Montgomery	
Enquist, Brian	Pevzner, Pavel	
Enright, Anton	Quattrone, Alessandro	
Feingold, Mario	Raval, Alpan	
Fetrow, Jacquelyn	Robertson, David	
Friedberg, Iddo	Ross, Elliot	
Garcia Martin, Hector	Roux, Benoit	
Gimble, Jeffrey	Sauer, Uwe	
Goodson, Holly	Schlick, Tamar	
Harrison, Paul	Schultz, Simon	
Hartemink, Alexander	Sept, David	

PLoS Computational Biology Reviewers 2008

Aach, John	Arganda, Sara	Becskei, Attilla	Breakspear, Michael
Abeles, Moshe	Arstila, T. Petteri	Beer, Michael	Brennan, Michael
Adami, Chris	Asai, Kiyoshi	Beer, Randy	Bourret, Robert
Adams, Mark	Asimenos, George	Beerenwinkel, Niko	Bower, James
Aebi, Ueli	Asquith, Becca	Beggs, John	Bowman, Howard
Aggarwal, Amit	Assisi, Collins	Benfey, Philip	Braitenberg, Valentin
Agrawal, Aneil	Atilgan, Canan	Benham, Craig	Brenner, Eli
Agur, Zvia	Atreya, Hanudatta	Bennett, Max	Brenner, Michael
Akey, Joshua	Attwell, David	Benos, Takis	Brent, Doiron
Akke, Mikael	Audinat, Etienne	Ben-Tal, Nir	Brent, Michael
Ala-Korpela, Mika	Averbeck, Bruno	Berezovsky, Igor	Bressloff, Paul
Alberghina, Lilia	Ay, Nihat	Berger, Bonnie	Briggs, James
Albert, Istvan	Azevedo, Ricardo	Bergeron, Anne	Brown, Duncan
Albert, Reka	Babu, Madan	Bergman, Casey	Bruggeman, Frank
Albrecht, Mario	Babu, Suresh	Bergmann, Sven	Brunel, Nicolas
Aldana, Maximino	Bader, Joel	Berkes, Pietro	Brusic, Vladimir
Al-Hasani, Hadi	Badger, Jonathan	Bernacchia, Alberto	Bryson, Kevin
Alizon, Samuel	Bafna, Vineet	Bernauer, Julie	Bucher, Philipp
Allen, Timothy	Bahar, Ivet	Bertram, Richard	Bullmore, Ed
Alm, Eric	Bailey, Timothy	Betel, Doron	Bundschuh, Ralf
Almaas, Eivind	Baitaluk, Michael	Bettencourt, Luis	Buonomano, Dean
Almonacid, Daniel	Baker, Nathan	Beyer, Andreas	Burch, Christina
Aloy, Patrick	Balasubramaniam, Ramesh	Bi, Guo-Qiang	Burgess, Neil
Alt, Wolfgang	Balazsi, Gabor	Bild, Andrea	Burkom, Howard
Alter, Orly	Bandelt, Hans-Jürgen	Biondini, Mario	Burton, Mike
Althaus, Christian	Bansal, Shweta	Bjaalie, Jan	Bussemaker, Harmen
Alvarez-Buylla, Elena	Bao, Zhirong	Bocharov, Gennady	Butler, Peter
Amaral, Luis	Barad, Omer	Bock, Christoph	Butts, Daniel
Amirikian, Bagrat	Barash, Danny	Boelle, Pierre-Yves	Bystroff, Chris
An, Gary	Bar-Joseph, Ziv	Bolshoy, Alexander	Caetano-Anolles, Gustavo
Anastassiou, Dimitris	Barkai, Naama	Boni, Maciej	Caflisch, Amedeo
Anderson, Sandy	Barlow, Dave	Bonneau, Richard	Cagney, Gerard
Andrade, Miguel	Barton, Nick	Bootsma, Martin	Calvetti, Daniela
Andrade-Navarro, Miguel	Bastiaens, Philippe	Borenstein, Elhanan	Camacho, Carlos
Andras, Peter	Basu, Malay	Borghans, Jose	Camerer, Colin
Andreasen, Viggo	Batchelor, Eric	Borgwardt, Karsten	Caminiti, Roberto
Andrews, Steven	Bates, Paul	Borisyuk, Alla	Campagne, Fabien
Andrews, Steven	Batzoglou, Serafim	Bornholdt, Stefan	Cannon, Robert
Andricioaei, Ioan	Bauch, Chris	Borst, Alexander	Carlson, Heather
Antes, Iris	Baumketner, Andriy	Bossaerts, Peter	Carlsson, Anders
Antia, Rustom	Bazhenov, Maxim	Botvinick, Matthew	Carmi, Shai
Arenas, Alex	Beard, Daniel	Boumpas, Dimitrios	Carpenter, Anne
Ares Jr., Manuel	Beck, Jeff	Bourque, Guillaume	Casadio, Rita

Castillo-Davis, Cristian	Cowan, Jack	Di Chialvo, Dante	Evans, James
Cauchemez, Simon	Cowan, Noah	Di Ventura, Barbara	Eyras, Eduardo
Centola, Mike	Cowling, Benjamin	Diedrichsen, Jörn	Faeder, Jim
Ceruso, Marco Ceruso	Cowperthwaite, Matt	Diesmann, Markus	Faisal, Aldo
Cesareni, Gianni	Craciun, Gheorghe	Dietrich, Fred	Fares, Mario
Chakraborty, Arup	Crandall, Keith	Dimitrov, Alex	Fariselli, Piero
Chalub, Fabio	Crauste, Fabien	Ding, Mingzhou	Fast, Naomi
Chan, Christina	Craze, Paul	Ditterich, Jochen	Faugeras, Olivier
Chandran, Deepak	Crow, Mary	Dixit, Narendra	Fazl, Arash
Chang, Howard	Csikasz-Nagy, Attila	Do, Chuong	Fedorov, Alexei
Cheatham, Tomas E	Cui, Qiang	Dolan, Raymond	Fedorova, Natalie
Chen, Irene	Cui, Yuehua	Domany, Eytan	Feenstra, Anton
Chen, Yi-Ping Phoebe	Cuntz, Hermann	Donahue, Leah Rae	Feldgarden, Michael
Chennubhotla, Chakra	Czirók, András	Donald, Bruce	Feldman, Marcus
Chialvo, Dante	Daily, Michael	Donaldson-Matasci, Matina	Fell, David
Chitnis, Ajay	Daunizeau, Jean	Donkelaar, C.C. van (René)	Feng, Jianfeng
Chizhov, Anton	Davenport, Miles	Dosztyani, Zsuzsanna	Fetrow, Jacquelyn
Chklovskii, Dmitri	David, Olivier	Dougherty, Daniel	Fiete, Ila
Chodera, John	Davidich, Maria	Dowse, Harold	Fink, J. Lynn
Chou, Tom	Davidson, Lance	Doyle, Frank	Finkenstadt, Barbel
Chow, Carson	Davis, Fred	Draguhn, Andreas	Fischer, William
Choy, James	Davis, George	Drummond, Alexei	Fiser, Andras
Christodoulou, John	Daw, Nathaniel	Drummond, Allan	Fisher, Daniel
Chrobak, James	Day, Troy	Duan, Yong	Fisher, Jasmin
Chung, Wen-Yu	Dayan, Peter	Dumontier, Michel	Fisher, Paul
Cinquin, Olivier	De Boer, Rob	Dunbrack, Roland	Flucher, Bernhard
Ciupe, Stanca	De Groot, Anne	Dushoff, Jonathan	Flyvbjerg, Henrik
Civelli, Olivier	De Lichtenberg, Ulrik	Ebenhöh, Oliver	Földiák, Peter
Clifford, Colin	De Los Rios, Paolo	Eddy, Sean	Fong, Stephen
Cline, Melissa	De Schutter, Erik	Edelstein-Keshet, Leah	Fontana, Walter
Cohen, Barak	De Vries, Gerda	Egert, Ulrich	Fortune, Eric
Cokol, Murat	De Zeeuw, Chris	Ehrenberg, Måns	Fouchet, David
Collins, James	Deane, Charlotte	Einhaeuser, Wolfgang	Fraenkel, Ernest
Collins, Lesley	Deckard, Anastasia	Eisen, Jonathan	Frank, Loren
Collins, Sean	Deco, Gustavo	Eisen, Michael	Fraser, Christophe
Colman-Lerner, Alejandro	DeFroment, Adrian	Elf, Johan	Fraser, Hunter
Compte, Albert	DeLuca, David	Ellington, Andrew	Freilich, Shiri
Conradi, Carsten	DeLuna, Alexander	Elofsson, Arne	Frenkel, Daan
Coombes, Steve	Den Blaauwen, Tanneke	Emonet, Thierry	Frey, Brendan
Coombs, Dan	Dennis, Jonathan	Endres, Robert	Fried, Susan
Cooper, Gregory	Derreumaux, Philippe	Erdi, Peter	Friedberg, Iddo
Corron, Ned	DeSchutter, Erik	Erives, Albert	Friedman, Nir
Cortassa, Sonia	Dessailly, Benoit	Ermentrout, Bard	Friedman, Nir
Cote, Helene	Destexhe, Alain	Eskin, Eleazar	Frigessi, Arnoldo
Coutinho, Evans	Deutsch, Andreas	Esposito, Luciana	Frisen, Jonas
Covert, Markus	Di Bernardo, Diego	Evans, Ben	Frishman, Dmitrij

Froemke, Robert	Gonze, Didier	Hecht, Michael	Humphreys, Glyn
Froenicke, Lutz	Gorodkin, Jan	Heckerman, David	Husmeier, Dirk
Frost, Simon	Gough, Julian	Hedrick, Tyson	Huss, Mikael
Frye, Mark	Goulian, Mark	Hein, Jotun	Hutt, Axel
Fukai, Tomoki	Graham, Bruce	Heinemann, Matthias	Huynen, Martijn
Furey, Terrence	Gramada, Apostol	Heinke, Dietmar	Huys, Quentin
Fusi, Stefano	Granseth, Erik	Hellgren Kotaleski, Jeanette	Hwang, Wonmuk
Fuxreiter, Monika	Grant, Barry	Helmer-Citterich, Manuela	Iakoucheva, Lilia
Gabaldón, Toni	Graupner, Michael	Helms, Volkhard	Ideker, Trey
Gabbiani, Fabrizio	Gray, Jeffrey	Hensel, Michael	Idiart, Marco
Gaedke, Ursala	Griesinger, Christian	Henson, Michael	Igoshin, Oleg
Galán, Roberto	Griffiths, Timothy	Hermann, Torsten	Ikegaya, Yuji
Galitski, Timothy	Griffiths-Jones, Sam	Herschlag, Daniel	Iliopoulos, Costas
Gallant, Jack	Grishin, Nick	Hertel, Jana	Imoto, Seiya
Galperin, Michael	Gromiha, Michael	Hertel, Klemens	Indic, Premananda
Galtier, Nicolas	Grossfield, Alan	Herzel, Hanspeter	Ingalls, Brian
Galvani, Alison	Grunbaum, Daniel	Higgs, Paul	Intaglietta, Marcos
Galzitskaya, Oxana	Guarnieri, Frank	Hilfinger, Andreas	Ionides, Ed
Gandon, Sylvain	Guigon, Emmanuel	Hilgenfeldt, Sascha	Ioshikhes, Ilya
Ganusov, Vitaly	Gulbahce, Natali	Hiller, Michael	Iossifov, Ivan
Gao, Mu	Gunderson, Samuel	Hintze, Arend	Isaacs, Farren
Garcia-Ojalvo, Jordi	Gupta, Mayetri	Hirschman, Lynette	Ishii, Shin
Garde, Shekhar	Gupta, Sunetra	Hishiki, Teruyoshi	Itoh, Satoru
Gardner, Daniel	Gursoy, Attila	Hoch, Jeff	Itoh, Takeshi
Gardner, Paul	Guthrie, Christine	Hoefer, Thomas	Itzkovitz, Shalev
Gat-Viks, Irit	Gutkin, Boris	Hofacker, Ivo	Iyer, Vishy
Gavrilets, Sergey	Haas, Julie	Hohn, Thomas	Jaakkola, Tommi
Gerland, Ulrich	Hahnloser, Richard	Holden, James	Jack, Thomas
Gerstner, Wulfram	Hakim, Vincent	Holderied, Marc	Jackson, Sophie
Getz, Wayne	Hammer, Daniel	Holm, Liisa	Jacobs, Chris
Ghani, Azra	Hampson, Robert	Holmes, Ian	Jacobson, Matthew
Ghazanfar, Asif	Han, Jing-Dong	Holmes, William	Jaeger, Johannes
Ghilardi, M. Felice	Hanada, Kousuke	Holzhütter, Hermann-Georg	Jafri, Saleet
Giaume, Christian	Handel, Andreas	Hong, Fangxin	Janes, Kevin
Gibb, Alasdair	Hannenhalli, Sridhar	Honig, Barry	Janga, Sarath
Gielen, Stan	Hardy, Jeanne	Hopfield, John	Janin, Joel
Gintis, Herbert	Hardy, Richard	Horn, Richard	Jauregui, Ruy
Girolami, Mark	Hartemink, Alexander	Horvath, Steve	Jayaprakash, Ciriyam
Goffaux, Valérie	Hartzell, Patricia	House, Thomas	Jbabdi, Saad
Gojobori, Takashi	Hasenstaub, Andrea	Hoyos, Mauricio	Jeffries, Thomas
Golding, Ido	Hasselmo, Michael	Hrabe, Jan	Jensen, Lars
Goldman, Mark	Hasty, Jeff	Hruby, Victor	Jensen, Mark
Goldstein, Richard	Hatzigeorgiou, Artemis	Hubbard, Tim	Jensen, Thomas Skott
Golomb, David	Hatzimanikatis, Vassily	Hucka, Mike	Jernigan, Robert
Goltsman, Eugene	Haugh, Jason	Hughes, Austin	Jirsa, Viktor
Gomez, Shawn	He, Yongqun	Hulsen, Tim	John, Bino
Johnson, Kenneth	Kishony, Roy	Lahav, Galit	Lise, Stefano
Jolivet, Renaud	Kitano, Hiroaki	Landau, Emmanuel	Lisman, John

Jung, Peter	Klamt, Steffen	Lappe, Michael	Listgarten, Jennifer
Jurisica, Igor	Kleywegt, Gerard	Laskowski, Roman	Littlewood, Peter
Kording, Konrad	Klimov, Dmitri	Lassig, Michael	Liu, Jun
Kaern, Mads	Klingmüller, Ursula	Lau, Brian	Liu, Xiaole
Kafri, Ran	Klipp, Edda	Laub, Michael	Liu, Zhirong
Kaiser, Marcus	Knight, Rob	Laughlin, Simon	Lloyd-Smith, James
Kalinina, Olga	Knill, David	Laughton, Charles	Lobley, Anna
Kalisky, Tomer	Koehl, Patrice	Lavery, Richard	Lockless, Steve
Kamburov, Atanas	Koella, Jacob	Le Roch, Karine	Loewenstein, Yonatan
Kamm, Roger	Koelle, Katia	Lecar, Harold	London, Mickey
Kannan, Natarajan	Koenig, Peter	Lee, Andrew	Long, Manyuan
Kanwisher, Nancy	Kontaxis, Georg	Lee, BK	Lopez, Luis
Kaplan, Shai	Koonin, Eugene	Lee, Daeyeol	Lopez-Poveda, Enrique
Kaplan, Tommy	Korber, Bette	Lee, Jeannie	Louis, Edward
Karbowski, Jan	Kording, Konrad	Lee, Sang Yup	Louis, Matthieu
Karlsson, Andreas	Korf, Ian	Lee, Tai Sing	Louzoun, Yoram
Karniel, Amir	Kornblihtt, Alberto	Lee, Tim	Lovell, Simon
Karpievitch, Yuliya	Kortemme, Tanja	Lee, William	Lowe, Todd
Kass, Robert	Kosakovsky Pond, Sergei	Leergard, Trygve	Loytynoja, Ari
Kaufman, Jim	Kötter, Rolf	Leibold, Christian	Lu, Hui
Kavraki, Lydia	Kouyos, Roger	Leitner, Thomas	Lu, Jian
Kaznessis, Yiannis	Koza, Robert	Leloup, Jean-Christophe	Lu, Yong
Keasar, Chen	Krakauer, David	Lengyel, Máté	Lubbell, William
Keasling, Jay	Krakauer, John	Leslie, Christina	Luisi, Pier
Keating, Amy	Krallinger, Martin	Levchenko, Andre	Lungarella, Max
Keles, Sunduz	Krauss, Scott	Levin, Matthew	Lunter, Gerton
Kellis, Manolis	Krauthammer, Michael	Levine, Erel	Lupas, Andrei
Kelly, John	Krebs, Hermano	Levine, Herbert	Müller, Viktor
Kelso, Janet Scott	Kreitman, Martin	Levy, Ronald	Ma, Jianpeng
Kemp, Graham	Krichmar, Jeffrey	Levy, Yaakov	Mac Gabhann, Feilim
Kepler, Thomas	Krishnan, J	Lewicki, Mike	Machens, Christian
Kerkhoff, Andrew	Kriwacki, Richard	Lewis, Tim	Macias, Maria
Kersten, Dan	Krogan, Nevan	Leyser, Ottoline	MacIssac, Kenzie
Kesmir, Can	Krogh, Anders	Li, Lei	Madura, Jeffry
Khosla, Chaitan	Kruger, Warren	Li, Yi-Xue	Mahadevan, Radhakrishnan
Kiebel, Stefan	Krukenberg, Kristin	Liang, Shoudan	Mak, Huajiang Craig
Kiehart, Daniel	Kuhara, Satoru	Liao, Li	Maloney, Laurence
Kiel, Christina	Kuhn, Jeffrey	Liberles, David	Mamitsuka, Hiroshi
Kilpatrick, A. Marm	Kumar, Sudhir	Lichtarge, Olivier	Mancilla, Jaime
Kim, Peter	Kummer, Ursula	Liley, David	Maranas, Costas
Kim, Philip	Kuo, Chung-Chin	Lin, Baochuan	Maranas, Costas
Kimmel, Alan	Kurths, Jürgen	Lindow, Morten	Maravall, Miguel
Kimmel, Marek	Ladbury, J. E.	Lipkow, Karen	Marcotte, Edward
Kinzer-Ursem, Tamara	Lagergren, Jens	Lipsitch, Marc	Marenduzzo, Davide
Margolin, Adam	Moses, Alan	Olsson, Bjorn	Penny, William
Margolis, Elisa	Mosier, Donald	Olufsen, Mette	Peregrín-Alvarez, José Manuel

Maritan, Amos	Moya, Andres	Oprea, Tudor	Pereira-Leal, Jose
Markel, Scott	Muller, Rolf	Oren, Aharon	Perelson, Alan
Martin, Bill	Mundy, Chris	Orengo, Christine	Perrier, Michel
Martin, Pascal	Munro, Edwin	Ostman, Bjorn	Pesole, Graziano
Martins dos Santos, Vitor	Murray, Douglas	Othmer, Hans	Pessiglione, Mathias
Masel, Joanna	Mushegian, Arcady	Ouzounis, Christos	Peters, Bjoern
Maslov, Sergei	Myers, Chad	Ovcharenko, Ivan	Pfeiffer, Dirk
Mathews, David	Nadal, Jean-Pierre	Ozer, Mahmut	Phillips, Andrew
Mayo, Avraham	Naef, Felix	Oztop, Erhan	Pillai, Satish
McConkey, Glenn	Nakagaki, Toshiyuki	Pál, Csaba	Pilyugin, Sergei
Mcdermott, Jason	Nanda, Santosh	Pachter, Lior	Pinney, John
McIntosh, Randy	Näsänen, Risto	Paci, Emmanuele	Pinter, Ron
McLaughlin, Tracey	Neafsey, Daniel	Pacold, Mary	Pipa, Gordon
McLysaght, Aoife	Neill, Daniel	Padinhateeri, Ranjith	Pitkow, Xaq
Mehr, Ramit	Nelken, Israel	Pagel, Philipp	Plenz, Dietmar
Mehring, Carsten	Nemenman, Ilya	Pai, Dinesh	Poenie, Martin
Meir, Ron	Neshich, Goran	Paixao, Tiago	Poirazi, Panayiota
Meir, Yigal	Netoff, Theoden	Pal, Csaba	Polidori, David
Mel, Bartlett	Nettleton, Daniel	Palczewski, Krzysztof	Pombo, Ana
Merz, Keth	Newman, Stuart	Palli, Subba	Poole, Leslie
Meyer, Irmtraud	Nguyen, Liem	Panchenko, Anna	Posada, David
Meyer-Hermann, Michael	Nieder, Andreas	Pande, Vijay	Pouget, Alexandre
Micheletti, Cristian	Nielsen, Jens	Panzeri, Stefano	Poupon, Anne
Micheyl, Christophe	Nielsen, Morten	Papageorgiou, Lazaros	Powers, Robert
Michod, Rick	Nikolau, Christoforos	Papin, Jason	Prabhakar, Shyam
Miller, Crispin	Nilges, Michael	Papoian, Garegin	Pressnitzer, Daniel
Miller, John	Nirenberg, Sheila	Papp, Balázs	Price, Nathan
Minayev, Pavlo	Nislow, Corey	Pappu, Rohit	Print, Cristin
Mindlin, Gabriel	Niv, Masha	Parga, Néstor	Prinz, Astrid
Minor, Dan	Noble, William	Park, Woong-Yang	Proulx, Stephen
Mirny, Leonid	Noe, Frank	Parkhill, Julian	Prusinkiewicz, Przemyslaw
Misevic, Dusan	Noskov, Sergei	Parmeggiani, Andrea	Przulj, Natasa
Mitchell, Julie	Notebaart, Richard	Passingham, Richard	Przytycka, Teresa
Miyano, Satoru	Novak, Bela	Pastore, Annalisa	Ptitsyn, Andrey
Mizrahi, Adi	Nowotny, Thomas	Paten, Benedict	Pugh, Frank
Mjolsness, Eric	Nussinov, Ruth	Patterson, Nick	Punta, Marco
Moeller, Steffen	O'Carroll, David	Pavlidis, Paul	Quackenbush, John
Mogilner, Alex	Odde, David	Pawelzik, Klaus	Quiroga, Rodrigo Quian
Mollicone, Daniel	O'Doherty, John	Pe'er, Itsik	Rabadan, Raul
Monk, Nick	O'Dwyer, James	Peirce, Shayn	Rabinovich, Misha
Montague, Read	Ofran, Yanay	Peixoto, Natalia	Rabinowitz, Joshua
Morais-Cabral, Joao	Ohler, Uwe	Peled, Avraham	Rader, Andrew
Moran, Rosalyn	Okamoto, Yuko	Pellegrini, Matteo	Radivojac, Predrag
Morgansen, Kristi	Oliva, Baldomero	Pellegrini-Calace, Marialuisa	Raghavan, Raghu

Ramnani, Narender	Roxin, Alexander	Senn, Walter	Solé, Ricard
Rand, David	Roy, Krishnendu	Serrano, Luis	Sompolinsky, Haim
Ranganathan, Rama	Roy, Shovonlal	Seymour, Ben	Song, Yun
Rao, Christopher	Ruan, Jianhua	Shah, Mala	Soong, Ta-tsen
Ravasi, Timothy	Rubin, Jonathan	Shakhnovich, Boris	Sorg, Christian
Ray, Animesh	Ruediger, Stefan	Shakhnovich, Eugene	Sorger, Peter
Ray, J	Ruppin, Eytan	Shalem, Ophir	Sorin, Eric
Rebhan, Michael	Rusakov, Dmitri	Shalgi, Reut	Sorribas, Albert
Rebholz-Schuhmann, Dietrich	Russell, Colin	Sharan, Roded	Soyer, Orkun
Recchia, Angela	Ryu, William	Sharpee, Tatyana	Spang, Rainer
Recker, Mario	Sachidanandam, Ravi	Shea-Brown, Eric	Sporns, Olaf
Redelings, Benjamin	Sachs, Jonathan	Shearwin, Keith	Spouge, John
Redish, David	Sachse, Frank	Shendure, Jay	Srinivasan, Balaji
Reed, Jennifer	Saenko, Evgueni	Shen-Orr, Shai	Stadler, Michael
Regehr, Wade	Sahani, Maneesh	Shera, Christopher	Stadler, Peter
Regoes, Roland R.	Sahinalp, Cenk	Sherlock, Gavin	Stajich, Jason
Reichardt, Jörg	Sajikumar, Sreedharan	Shieh, Grace	Stam, Cornelis
Reinert, Knut	Salathe, Marcel	Shimizu, Kazuyuki	Stanke, Mario
Reinkensmeyer, David	Salsbury, Jr, Freddie	Shimizu, Tom	Stark, Alexander
Remez, Robert	Samoilov, Michael	Shin, Seokmin	Stark, Scott
Remme, Espen	Sampath, Ranga	Shinar, Guy	Stelling, Jorg
Renart, Alfonso	Samuel, Aravinthan	Shoichet, Brian	Stephan, Klaas
Ribas de Pouplana, Luis	Sander, Oliver	Shokat, Kevan	Steuer, Ralf
Rich, Thomas	Santillan, Moises	Shvartsman, Stanislav	Stiles, Joel
Riera, Jorge	Sarkar, Casim	Siepel, Adam	Stolovitzky, Gustavo
Rigoutsos, Isidore	Sauer, Uwe	Sigmund, Karl	Stormo, Gary
Rinberg, Dmitry	Sauro, Herbert	Silander, Olin	Stramaglia, Sebastiano
Rind, Claire	Schächter, Vincent	Simon, Itamar	Strimmer, Korbinian
Ringe, Dagmar	Schaefer, Carl	Simon, Melvin	Stuart, Josh
Rinzel, John	Schäfer, Jürgen	Singh, Amoolya	Sudol, Marius
Rivas, Elena	Schaffer, David	Singh, Harinder	Sumpter, David
Rivoire, Olivier	Schiff, Nicholas	Singh, Mona	Sundquist, Andreas
Roach, Jared	Schiff, Steven	Sinha, Saurabh	Sunkar, Ramanjulu
Robinson, Hugh	Schlessinger, Avner	Sizling, Arnost	Supp, Gernot
Robinson, Peter	Schlicker, Andreas	Sjolander, Kimmen	Surmeier, D.
Robinson-Rechavi, Marc	Schmucker, Dietmar	Slonim, Donna	Sutherland, Jeff
Robison, Keith	Schnell, Santiago	Smalheiser, Neil	Swain, Peter
Rodrigo, Allen	Scholz, Matthias	Small, Stephen	Swenson, Krister
Rognan, Didier	Schrater, Paul	Smilde, Age	Swindale, Nicholas
Rogozin, Igor	Schuster, Stefan	Smith, Barry	Szathmáry, Eörs
Rolls, Edmund	Schwartz, Andrew	Smith, Derek	Szatmary, Botond
Ronson, Clive	Schwartz, Odelia	Smolke, Christina	Sze, Sing-Hoi
Rost, Burkhard	Schyns, Philippe	Sneppen, Kim	Szilágyi, Andras
Roth, Fritz	Segal, Eran	Snoeyink, Jack	Taatjes, Dylan
Roudi, Yasser	Segre, Daniel	Sobie, Eric	Tabak, Joel
Roux, Benoit	Sejnowski, Terrence	Sogaard-Andersen, Lotte	Tajhkorshid, Emad

Tamames, Javier	Trifonov, Edward	Vervaeke, Koen	Wheelan, Sarah
Tanay, Amos	Trommershaeuser, Julia	Vicsek, Tamas	White, Forest
Tang, Guiliang	Troyanskaya, Olga	Vida, Imre	White, Michael
Tang, Jon	Tsai, Jerry	Vidal, Marc	Wiggins, Chris
Tani, Jun	Tsoka, Sophia	Vilfan, Andrej	Wilbur, John
Tanzer, Andrea	Tsuda, Koji	Vingron, Martin	Wilcken, Bridget
Tarek, Mounir	Tsunoda, Tatsuhiko	Viola, Haydée	Wiley, H. Steven
Tartaglia, Gian	Tu, Benjamin	Vishveshwara, Saraswathi	Wilke, Claus
Tass, Peter	Tucker-Kellogg, Greg	Vitkup, Dennis	Wilson, Derek
Taubenberger, Jeffery	Tucker-Kellogg, Lisa	Vodovotz, Yoram	Winn, Philipp
Taylor, Martin	Turner, Ray	Vogt, Richard	Winslow, Raimond
Taylor, Susan	Tusnady, Gabor E	Voit, Eberhard	Wiskott, Laurenz
Teeling, Hanno	Tyson, John	Von Mering, Christian	Wittenberg, Gayle
Ten Wolde, Pieter Rein	Ulanovsky, Nachum	Wade, Rebecca	Wittinghofer, Fred
Ter Keurs, Henk	Umulis, David	Wagner, Andreas	Wodak, Shoshana
Terry, John	Uversky, Vladimir	Wagner, Günter	Wong, Chung
Tesler, Glenn	Vaishnavi, Neil	Wall, Judy	Wong, Limsoon
Teusink, Bas	Vaisman, Iosif	Wall, Michael	Wootton, John C.
Thanaraj, Alphonse	Vajda, Sandor	Wallin, Stefan	Wörgötter, Florentin
Thirumalai, Dev	Vaknin, Ady	Wallinga, Jacco	Wu, Song
Thomas, Dave	Vakser, Ilya	Wallis, Guy	Xie, Lei
Thomas, Kelley	Valdes-Sosa, Pedro	Wallstrom, Garrick	Xing, Yi
Thomas, Serge	Valencia, Alfonso	Walz, Wolfgang	Xu, Dong
Thompson, Mark	Valiente, Gabriel	Wang, Ing-Nang	Xu, Huafeng
Thorne, Jeffrey	Valouev, Anton	Wang, Jin	Yaeger, Larry
Tiana, Guido	Van Boven, Michiel	Wang, Kai	Yakhini, Zohar
Tibshirani, Rob	Van der Oost, John	Wang, Wei	Yandell, Brian
Tidor, Bruce	Van der Vaart, Arjan	Wang, Xiao-Jing	Yang, Feng
Tiesinga, Paul	Van Dijk, Pim	Wang, Zefeng	Yates, Andrew
Timmer, Jens	Van Driel, Roel	Warner, Digby	Ye, Ping
Timofeev, Igor	Van Noort, Vera	Wass, Mark	Yeger-Lotem, Esti
Tipton, Peter	Van Oudenaarden, Alexander	Wearne, Susan	Yeo, Christopher
Tirado-Rives, Julian	Van Rossum, Mark	Webb, Alex	Yeo, Gene
Tjaden, Brian	Van-Vreeswijk, Carl	Wehrle-Haller, Bernhard	Yi, Tau-Mu
Todorov, Emo	Varani, Gabriele	Wei, Guanghong	Yildirim, Muhammed
Tompa, Peter	Varner, Jeffrey	Weijer, Cornelis	Yin, John
Tononi, Giulio	Vavylonis, Dimitrios	Weinstein, Harel	Yomo, Tetsuya
Torrents, David	Vazquez, Alexei	Weir, Bruce	York, William S.
Torres, Joaquin	Vendruscolo, Michele	Weiss, Ron	Yoshida, Wako
Tosatto, Silvio	Ventura, Salvador	Weiss, Yair	You, Lingchong
Toyoizumi, Taro	Venturi, Venessa	Weitz, Joshua	Young, Martin
Traulsen, Arne	Veretnik, Stella	Weliky, Michael	Yu, Angela
Traynelis, Stephen	Verkhivker, Gennady	Wennekers, Thomas	Yugi, Katsuyuki
Treves, Alessandro	Vert, Jean-Philippe	Westbrook, John	Yusim, Karina

Zacharias, Martin
Zamore, Phillip
Zapotocky, Martin
Zecchina, Riccardo
Zhang, Jinghui
Zhang, Kechen
Zhang, Weixiong
Zhang, Xuegong
Zhang, Yang
Zhang, Zhaolei
Zhao, Hongyu
Zhao, Keji
Zhaoping, Li
Zheng, Jie
Zheng, Wenjun
Zheng, Yun
Zhong, Sheng
Zhou, Huan-Xiang
Zhou, Jasmine
Zhou, Ruhong
Zhou, Xianghong
Zhou, Xuefeng
Zhulin, Igor
Zhuo, Min
Zimmer, Ralf
Zocchi, Giovanni
Zotenko, Elena
Zuidema, Willem
Zupan, Blaz
